# Reporting of complex interventions in clinical trials: development of a taxonomy to classify and describe fall-prevention interventions

**DOI:** 10.1186/1745-6215-12-125

**Published:** 2011-05-17

**Authors:** Sarah E Lamb, Clemens Becker, Lesley D Gillespie, Jessica L Smith, Susanne Finnegan, Rachel Potter, Klaus Pfeiffer

**Affiliations:** 1 Warwick Clinical Trials Unit, University of Warwick, Gibbet Hill Campus, Coventry, UK; 2 Kadoorie Critical Care Research Centre, John Radcliffe Hospital, University of Oxford, Headley Way, Oxford, UK; 3 Robert-Bosch-Hospital, Geriatric Rehabilitation Clinic, Auerbachstrasse 110, 70376 Stuttgart, Germany; 4 Department of Medicine, Dunedin School of Medicine, University of Otago, PO Box 913, Dunedin 9054, New Zealand

## Abstract

**Background:**

Interventions for preventing falls in older people often involve several components, multidisciplinary teams, and implementation in a variety of settings. We have developed a classification system (taxonomy) to describe interventions used to prevent falls in older people, with the aim of improving the design and reporting of clinical trials of fall-prevention interventions, and synthesis of evidence from these trials.

**Methods:**

Thirty three international experts in falls prevention and health services research participated in a series of meetings to develop consensus. Robust techniques were used including literature reviews, expert presentations, and structured consensus workshops moderated by experienced facilitators. The taxonomy was refined using an international test panel of five health care practitioners. We assessed the chance corrected agreement of the final version by comparing taxonomy completion for 10 randomly selected published papers describing a variety of fall-prevention interventions.

**Results:**

The taxonomy consists of four domains, summarized as the "Approach", "Base", "Components" and "Descriptors" of an intervention. Sub-domains include; where participants are identified; the theoretical approach of the intervention; clinical targeting criteria; details on assessments; descriptions of the nature and intensity of interventions. Chance corrected agreement of the final version of the taxonomy was good to excellent for all items. Further independent evaluation of the taxonomy is required.

**Conclusions:**

The taxonomy is a useful instrument for characterizing a broad range of interventions used in falls prevention. Investigators are encouraged to use the taxonomy to report their interventions.

## Background

Reduction of falls in older people is an important health care target in many countries. However, the aetiology of falls in older people is not fully understood. Although some falls result from a single factor, many are caused by an interaction between multiple factors [1]. As a consequence, a wide variety of approaches to prevention and management have emerged, most of which are "complex interventions" [2]. Complex interventions are usually described as interventions that contain several interacting components [2]. Despite many clinical trials which have identified effective interventions, translation into clinical practice has been slow [3]. There are many potential barriers to implementation of new interventions. Poor reporting of interventions in clinical trials can result in practitioners being unable to replicate the intervention. Researchers evaluating interventions in meta-analysis lack an internationally agreed framework for conceptualizing the main forms and important components of fall-prevention techniques, limiting the ability to draw meaningful comparisons and identify factors associated with success.

Several sets of international guidance have highlighted the importance of documenting and conceptualizing the influential components of an intervention, recognizing the importance of these steps in evaluation and understanding the potential for generalization of an intervention [4,5]. However, this documentation and conceptualization is not simple, particularly when trials may be from countries with different health and social care services.

The aim was to develop a classification system to characterize the major influential components of fall-prevention interventions and promote consistency of reporting across international boundaries. We wished to encourage investigators to report all fall prevention interventions in a standardised and comprehensive manner, and to produce a taxonomy that could be used to classify existing and future interventions. We report the process of development, involving repeated testing and refinement, from the initial stages of design through to the final version of the taxonomy.

The study was developed through the Prevention of Falls Network Europe (ProFANE) project, a collaborative project to reduce the burden of fall injury in older people through excellence in research and promotion of best practice [6]. The network activities were funded by the European Commission, but link clinicians, members of the public, and researchers worldwide.

## Methods

Classification systems are of two major types-typologies and taxonomies. Typologies are theoretically driven classification schemes. A taxonomy necessitates identifying characteristics that can be objectively measured in real-world circumstances, and have sufficient variance to demonstrate meaningful differences [7]. We aimed to develop an internationally accepted taxonomy. Sartorius [7] provides useful guidance on the development of taxonomies, highlighting the importance of broad agreement on the content and framework to ensure compliance with the intent and method of classification, and the need for an accompanying glossary defining operational terms. Our method was an iterative process, comprising a phase of development, followed by a period of refinement and finalisation. The process was informed and approved by international expert consensus throughout its span, the nature of the consensus building techniques varied during the project, but formal nominal group methods were implemented at key stages [8]. The consensus panel included 33 representatives of the academic, policy, practice, and user communities drawn from a range of disciplines and countries in Australasia, Europe and North America (detailed at the end of the manuscript). Disciplines included were medicine, physiotherapy, occupational therapy, nursing, psychology, public health, exercise physiology, statistics and epidemiology.

### Stage 1: Developing the taxonomy

**Figure 1:**
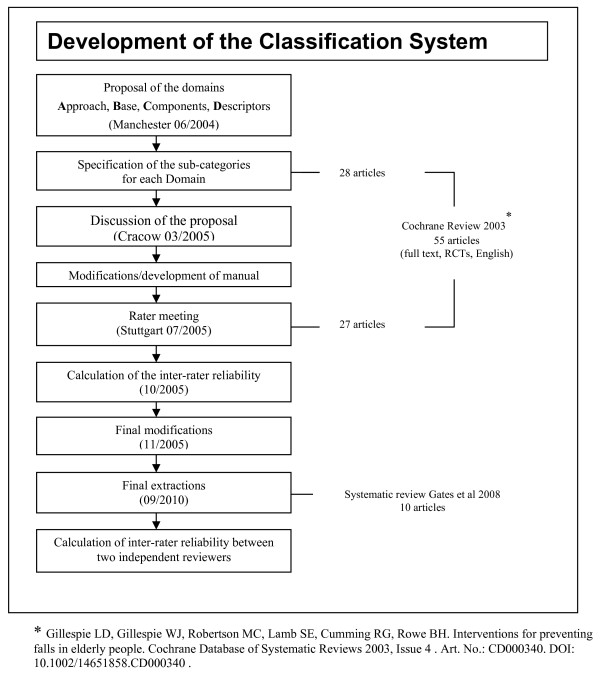
**Development of the Classification System**.

The process for developing the taxonomy is summarized in Figure 1. In the initial stages, a draft hierarchical framework of classification that identified the major domains was developed by a small group of investigators (SEL, EJS, KP, CB). This draft framework included the domain headings: "Approach", "Base", "Components" and "Descriptors". These domains provide the overall structure and framework to the taxonomy. The proposed framework was discussed and approved by a meeting of 50 academics and practitioners engaged in research in falls prevention in Manchester in 2004. Definitions were developed for each domain, along with sub-domains. The intention was that sub-domains would represent meaningful differences in the ways in which interventions had or could be delivered. Within each sub-domain the taxonomy was further characterized to provide more detailed levels of information on the service delivery model which we labeled as categories. The development was informed by close reference to the published literature to ensure the taxonomy was sufficiently comprehensive to describe the variety of interventions, settings and service delivery models reported in the research literature. Firstly, we identified all trials published in full and in English included in Gillespie et al [9], and used a random sampling method to select half of these (N = 28, [10-37]). We retained the remaining papers (N = 27) for validation studies in later components of the study. Second, we undertook a broader search of the non-randomised and grey literature search strategy reported in [38] to identify new or untested approaches to fall prevention. Where possible, we utilized existing international classification systems to provide category definitions and headings [39-43], and sourced additional definitions from the Medical Subjects Headings (MeSH) browser of the US National Library of Medicine. In some domains of the taxonomy, there were no pre-existing classification systems, and these were developed by consulting members of the expert panel, matching expertise to the areas requiring development. At each stage of the process, the rationale, definitions and instructions for completion of the various components of the taxonomy was documented, and turned into a draft instruction manual.

### Stage 2: Refining and agreeing the final version of the taxonomy and manual

The first version of the taxonomy and manual was circulated prior to a formal two-day consensus meeting of 33 international experts. We utilized a modified nominal group technique, facilitated by experienced health services researchers, in which panel members were asked to consider eight questions relating to the proposed taxonomy (shown in Table 1). The relevance of the domains, sub-domains and categories were agreed and prioritized by consensus panel. Only the most important categories were retained in the taxonomy, recognizing the need to balance detail against practical issues of completion and ease of communication. The panel also considered the future use of the taxonomy and its capacity to assimilate new and emerging areas of practice.

**Table 1:** Questions used to structure the modified nominal group.

1.	Can current interventions be included (i.e. is the taxonomy complete)?
2.	Is the proposed classification clear and meaningful? Does the typology include all potentially relevant and important factors, grouped in a meaningful manner?

3.	Is the identification of factors likely to be influential in determining clinical outcome, generalisability and implementation (i.e. the model) acceptable?

4.	Does the model adequately reflect the complexity of current interventions?

5.	Can future interventions be included in the model?

6.	Is the classification compatible with other classification systems?

7.	Is it feasible to report the required information in articles/reports/

The taxonomy and manual were then tested using the remaining 27 articles retained after our search of trials published in the Cochrane review [9,44-70]. Data on the intervention characteristics from these 27 papers were extracted by five academic health practitioners who had not been involved in development stages of the taxonomy (the test panel). These practitioners included disciplines of medicine, physiotherapy, occupational therapy, psychology and nursing. Each rater was provided with a copy of the taxonomy manual, and a recording form. In addition, a number of papers were replicated across the raters to enable estimation of the test re-test reliability of the taxonomy. A cut-off of poor agreement between raters (a cut of kappa <0.41 as per Landis and Koch [71]) was used to indicate areas of the taxonomy and manual requiring revision. We also worked with individual raters to determine areas of the manual and taxonomy that lacked clarity, were difficult to complete, or were incomplete. Results of the panel test were reported back to a group of 12 of the original members of the expert panel to agree the final format and manual for the taxonomy.

Finally, two independent reviewers completed a taxonomy for the interventions reported in ten randomised controlled trials selected at random from the systematic review of Gates et al [72-82], to determine the agreement and chance corrected agreement (kappa) for each sub-domain. Agreement was calculated over all categories included in each sub-domain, according to Landis and Koch [71]. Chance corrected agreement (kappa) was then established using SPSS (version 17) for each sub-domain for all ten papers. For each paper we then determined the frequency of items (kappa values) contained within each kappa banding (poor, fair, moderate, good or very good) as described by Altman [83]. The frequencies calculated were then added together across all ten papers and converted into a percentage. These results therefore show the percentage of items (kappa values) across all ten papers within each kappa banding.

## Results

The full taxonomy including the recording form and instruction manual are provided in Additional Files, 1 and 2. A description of the domains, sub-domains, and categories in each sub-domain are given in Table 2, along with a brief synopsis of the consensus justification for inclusion within the taxonomy. Agreement between the two independent raters for extraction for the final version of the taxonomy was good to excellent for all sub-domains. In over 90% of cases chance corrected agreement was very strong between the two independent raters (kappa = 0.81-1.00) (Table 3).

**Table 2:** Domains and sub-domains of the taxonomy of fall-prevention interventions

Domain	Sub-domain
Approach: describes the theoretical approach in terms of the primary aims, whether a population or individual approach has been used, and whether selection or targeting criteria have been used to identify cases or populations.	Primary aims of the intervention being developed Primary selection criteria used for case identification.

Base: describes where participants have been selected from, where the intervention is delivered and by whom	Recruitment or case identification: the site at which participants of the intervention were identified; Primary site of delivery: the site at which the majority of the intervention is delivered or targeted. Interventions delivered by : describes the individuals (professionals, trained professionals, etc) who deliver the majority of the intervention

Components: describes variations in assessments used for deciding treatments, the conceptual basis, and different methods of combining interventions	Assessments that are used Combination of interventions

Descriptors: describes each of the components delivered in the control including sub-classifications that are considered potentially important	Description of control group or sham interventions Description of the test interventions components

**Table 3:** kappa agreement for the final extractions by two raters

Kappa	Strength of agreement	Frequency (%)
0.81 - 1.00	Very Good	90.3

0.61 - 0.80	Good	2.1

0.41 - 0.60	Moderate	0.9

0.21 - 0.40	Fair	0.3

<0.20	Poor	6.5

## Discussion

The evaluation of complex interventions is an infant science. An inability to identify, define and communicate the potentially important components of falls prevention interventions has the potential to hinder development of research and uptake into clinical practice. We have identified key characteristics of interventions that we believe should be described alongside the publication of a randomised controlled trial of fall-prevention interventions. Many of the domains, sub-domains and categories are transferable to other similar interventions.

The taxonomy represents judgments in determining essential from non-essential information, and balances the need for brevity and simplicity against complexity and detail. There is no single accepted method to develop a taxonomy. Empirically based classification requires data, and in this case, data sources were publications of randomised controlled trials of fall-prevention interventions, meta-analyses and qualitative studies. This was supplemented by extensive involvement of experts, which is acknowledged as essential in developing taxonomies [84]. Elicitation of expert knowledge from consensus methods can be problematic [8]. We protected against predomination of individual's opinion by using a modified nominal group technique, guided by pre-specified questions and independent facilitation [8]. The modified nominal group technique was selected in preference to the Delphi method, as it allows discussion between experts to gain consensus [8].

The taxonomy went through several iterations, being refined by a diverse group of international experts at different time points. There were a number of internationally agreed classification systems already in existence, and we used these wherever possible. These included the controlled vocabulary (Medical Subject Headings (MeSH)) used to index MEDLINE. The performance of the draft taxonomy was assessed using retrospective data extraction from published papers. The validation sample contained a variety of intervention types. Whilst we have allowed for the possibility of new areas emerging within the taxonomy, further refinements will be required. For example, further refinement of the selection criteria section might include data on screening tools used, for example whether the tool intends to capture single or repeat fallers, or aims to identify osteoporotic fracture risk. We also accept that the sub-domains of the taxonomy may not be entirely distinctive in all situations, but that for the majority of situations it is fit for purpose. We intend to review the taxonomy in five years time and welcome feedback on the structure and content. 

The taxonomy is intended for a number of purposes, including to assist with data extraction alongside meta-analysis of research data, reporting of interventions tested in research studies, and in the process of development of interventions. The list is purposefully not exhaustive. The most recent update of the CONSORT guidelines for reporting clinical trials necessitates reporting on the main elements of complex interventions [5]. The challenges of reporting complex interventions have been recognised for sometime. The Medical Research Council (MRC) first published a framework for reporting and evaluating complex interventions in 2000 [85]. Although the MRC framework stresses the importance of accurate reporting, little operational guidance is provided. A recent suggestion has been to use graphical techniques to present key features of the timeline and content of interventions [4], a technique which can be expanded to a number of non-pharmacological interventions. In comparison, our method, which has more limited application as it focuses on fall-prevention interventions, is structured to ensure ease of complete reporting (by way of yes/no answers), uses standard internationally transferable set of definitions to describe the intervention components, settings and populations tested; and provides a more detailed description of intervention. Replication of interventions should be easier to achieve, although this assumption requires testing. We anticipate that similar methods could be developed in other fields. The taxonomy is complementary to but does not replace the Consort Guidance on the reporting of complex interventions [5].

Subsequently, the taxonomy has proved useful in a number of Cochrane and other high quality systematic reviews, and has enabled identification and pre-specification of important aspects of service configuration and intervention delivery [9,72,86-88]. We have also used the taxonomy as the framework for a UK national survey of falls services [3]. Authors of trials are encouraged to register details of their interventions using the taxonomy in a prospective format (open registration is available at http://www.warwick.ac.uk/go/fallstaxonomy), and to utilize the taxonomy to ensure accurate, complete and useful intervention reporting.

## Conclusion

We have developed a taxonomy to describe and classify fall-prevention interventions, with sufficient breadth to capture elements of the intervention that are thought influential in determining effectiveness. This was achieved through a mix of methods, including expert consensus, literature reviews, and validation by test panels.

## Competing interests

The authors declare that they have no competing interests.

## Authors' contributions

SL and CB had the concept for the original study, study design and analysis. SL wrote the first draft of the paper and is the guarantor. LG contributed search strategies and to the study design, analysis and write up. JS and SF were independent reviewers in final testing process and performed the statistical analysis. KP developed and edited the different versions of the taxonomy and manual, and contributed to the drafting of the manuscript. RP participated in developing the taxonomy. All authors read and approved the final version of the manuscript.

## Supplementary Material

Additional file 1 Manual for the fall prevention classification system Click here for file

Additional file 2 Taxonomy to describe and conceptualise fall prevention interventions Click here for file
