# Supplementary material for: Reporting of complex interventions in clinical trials: development of a taxonomy to classify and describe fall-prevention interventions
Source: Trials. 2011 May 17;12:125. doi: 10.1186/1745-6215-12-125 (PMC3127768; doi:10.1186/1745-6215-12-125)
Supplement: Additional file 1 — Manual for the fall prevention classification system [file 1745-6215-12-125-S1.DOC]

Taxonomy to describe and conceptualise fall prevention interventions **(Manual)**

(Lamb S, Becker C, Gillespie L, Smith J, Finnegan S, Potter R, Pfeiffer K on behalf of Taxonomy Investigators and the ProFaNE Group (www.profane.co))

5^th^ April 2007

Table of content

[Introduction 2](#__RefHeading___Toc266438351)

[Study reference & design 4](#__RefHeading___Toc266438352)

[Reference 4](#__RefHeading___Toc266438353)

[Design 4](#__RefHeading___Toc266438354)

[Domain 1: Approach 4](#__RefHeading___Toc266438355)

[Primary Aims 4](#__RefHeading___Toc266438356)

[Primary selection criteria 5](#__RefHeading___Toc266438357)

[Population approach 5](#__RefHeading___Toc266438358)

[Demographics 5](#__RefHeading___Toc266438359)

[Chronic diseases, symptoms, impairments 5](#__RefHeading___Toc266438360)

[Specific groups excluded 7](#__RefHeading___Toc266438361)

[Domain 2: Base 8](#__RefHeading___Toc266438362)

[Case Identification/Primary Site of Delivery 8](#__RefHeading___Toc266438363)

[Hospitals/Departments/Wards (Inpatient: Acute or Sub-acute) 8](#__RefHeading___Toc266438364)

[Nursing and residential care facilities (Non-acute) 9](#__RefHeading___Toc266438365)

[Providers of ambulatory health care 9](#__RefHeading___Toc266438366)

[Community based recruitment/delivery 11](#__RefHeading___Toc266438367)

[Assessments delivered by 12](#__RefHeading___Toc266438368)

[Interventions & postintervention follow-ups delivered by 13](#__RefHeading___Toc266438369)

[Professionals 13](#__RefHeading___Toc266438370)

[Trained non-professionals 14](#__RefHeading___Toc266438371)

[Domain 3: Components 16](#__RefHeading___Toc266438372)

[Assessment as part of the intervention (generic) 16](#__RefHeading___Toc266438373)

[Assessment as part of the intervention (specific) 16](#__RefHeading___Toc266438374)

[Combination 17](#__RefHeading___Toc266438375)

[Domain 4: Descriptors 18](#__RefHeading___Toc266438376)

[Exercises (supervised/unsupervised) 18](#__RefHeading___Toc266438377)

[Medication (Drug Target) 21](#__RefHeading___Toc266438378)

[Surgery 23](#__RefHeading___Toc266438379)

[Management of urinary incontinence 24](#__RefHeading___Toc266438380)

[Fluid or nutrition therapy 24](#__RefHeading___Toc266438381)

[Psychological 24](#__RefHeading___Toc266438382)

[Environment/Assistive technology 24](#__RefHeading___Toc266438383)

[Environmental (social environment) 26](#__RefHeading___Toc266438384)

[Knowledge 27](#__RefHeading___Toc266438385)

[Other interventions/procedures 27](#__RefHeading___Toc266438386)

[Further specifications of the intervention 27](#__RefHeading___Toc266438387)

Domain 4: [Descriptors/Control Group 29](#__RefHeading___Toc266438388)

[Supervised Exercises 29](#__RefHeading___Toc266438389)

[Medication(Drug Target) 29](#__RefHeading___Toc266438390)

[Social environment/Knowledge 29](#__RefHeading___Toc266438391)

[References. 30](#__RefHeading___Toc266438392)

# Introduction

The taxonomy is designed for three purposes

[1] To characterise and classify existing fall prevention interventions – such as those published in the literature as well as clinical services. It is being used by the Cochrane collaboration to characterise interventions, and by ProFaNE to map research activity to date and identify areas which need more research.

[2] To encourage authors of new interventions to report the intervention in such a way that it can be replicated and understood by others.

[3] To assist designers of new interventions to consider the range of factors that should be considered in developing and reporting a new intervention and to assist with pre-specification of a framework (model) explaining effectiveness for future testing.

You may be able to find more purposes for the taxonomy!

The study was developed by Workpackage 1 of the Prevention of Falls Network Europe project, a collaborative project to reduce the burden of fall injury in older people through excellence in research and promotion of best practice. ([www.profane.eu.org](http://www.profane.eu.org/)).

Tips on using the taxonomy

The taxonomy is divided into four domains ( see Table 1 for definitions). Within each domain there are further sub-domains (shown by grey headings in the taxonomy), and then finally within in each sub-domain, a further breakdown of categories. This manual provides a detailed description of the domains, sub-domains and categories.

Domains ⇒ Sub-domain ⇒ Category

The taxonomy is a balance between detailed description and a more reductionist approach. Occasionally it maybe difficult to find an exact sub-domain or category for the intervention you are detailing. This is most likely to occur beyond the sub-domain level. Our advice is to select the domains, sub-domains, and categories which best describe the intervention. In each sub-domain there is an section marked “other“ which allows you to enter any interventions that you cannot classify under the sub-domains currently available.

It is also important to recognise that the taxonomy has been designed for international comparison. For example, we have used the International Classification for Health Accounts to classify organisations who deliver healthcare. We would encourage you to fit your situation into this classification as best you can.

The taxonomy is complementary to but does not replace the Consort Guidance in the reporting of complex interventions.

Feedback

The taxonomy will evolve and grow over time. Would welcome any feedback to inform revisions to s.lamb@warwick.ac.uk

This manual should be read in conjunction with Lamb SE et al (Taxonomy paper).

Table 1. Domains and sub-domains of the ProFaNE taxonomy.

| **Domain 1: Approach: describes the theoretical approach in terms of the primary aims and whether and what selection or targeting criteria have been used to identify cases.**  **Sub-domains**  Primary aims of the intervention being developed  Primary selection criteria used for case identification.  **Domain 2: Base: describes where participants have been selected from, where the intervention is delivered and by whom**  **Sub-domains**  Recruitment site: the site at which participants of the intervention were identified;  Main site of delivery: the site at which the *majority* of the intervention is delivered or targeted.  Interventions delivered by : describes the individuals (professionals, trained professionals, etc) who deliver the majority of the intervention  **Domain 3: Components:** **describes variations in assessments used for deciding treatments, and different methods of combining interventions**  **Sub-domains:**  Assessments that are used as part of the intervention  Combination of interventions  **Domain 4: Descriptors: describes each of the components delivered in the control and active intervention, including sub-classifications that are considered potentially important**  **Sub-domains:**  Description of the test interventions components  Description of control group or sham interventions |
| --- |

# Domain 1: Approach

| Primary Aims | | |
| --- | --- | --- |
| To reduce falls (A100) | |  |
| To reduce fall related injuries (A101) | |  |
| To improve Quality of Life (A102) | | A generic concept reflecting concern with the modification and enhancement of life attributes, e.g., physical, political, moral and social environment; the overall condition of a human life. [MeSH D011788] |
| To improve function/physical activity (A103) | | e.g., mobility, body sway, balance etc. |
| To reduce hospitalisation/health care resource use (A104) | |  |
| Safety monitoring (A105) | | This category is likely to be used for retrospective data extraction from published studies only. It should be used where the primary aim of the intervention (listed under Others A199) tested was not to reduce falls, but falls were collected to monitor the safety of the intervention. |
| To improve psychological outcome (A106) | | Outcome measures targeting mental or behavioural characteristics of an individual or a group. (e.g. fear, self-efficacy, activity avoidance, loss of confidence) |
| Others (A199/A199A) | | All other primary aims not described under A100 to A106  A199A: Brief description (free text) |
| Primary selection criteria | | |
| Population approach (A200) | | These are approaches in which the entire population of older people are targeted. Examples are television media campaigns or mail shoot campaigns. No targeting criteria are specified, with the exception of age and gender (sometimes). |
| Selection criteria used | | |
| Demographics | | |
| Age group (A300) | | ≥ ….. years (Insert the minimum age of the participants according to the inclusion criteria) |
| Male only (A301) | |  |
| Female only (A302) | |  |
| Selected ethnic group (A303) | | Ethnic group: A group of people with a common cultural heritage that sets them apart from others in a variety of social relationships. [MeSH D005006].  If the inclusion and exclusion criteria for a study or programme did not state a specific ethnic group, but all members of the sample are from one ethnic group, then this box should not be ticked. |
| Others (A399/A399A) | | Not described under A300-A303  A399A: Brief description (free text) |
| Previous falls (≥ 1) (A400) | | At least one fall during the last year (self report or any records) |
| Chronic diseases, symptoms, impairments | | |
| Osteoporosis / osteoporotic (bone fragility) fractures (A500) | Osteoporosis: Reduction of bone mass without alteration in the composition of bone, leading to fractures. Primary osteoporosis can be of two major types: postmenopausal osteoporosis (OSTEOPOROSIS, POSTMENOPAUSAL) and age-related or senile osteoporosis. [MeSH D010024]  Osteoporosis, postmenopausal: Metabolic disorder associated with fractures of the femoral neck, vertebrae, and distal forearm. It occurs commonly in women within 15-20 years after menopause, and is caused by factors associated with menopause including estrogen deficiency. [MeSH D015663] | |
| Parkinson’s disease/ syndrome (A501) | Parkinson disease: A progressive, degenerative neurologic disease characterized by a TREMOR that is maximal at rest, retropulsion (i.e. a tendency to fall backwards), rigidity, stooped posture, slowness of voluntary movements, and a masklike facial expression. Pathologic features include loss of melanin containing neurons in the substantia nigra and other pigmented nuclei of the brainstem. LEWY BODIES are present in the substantia nigra and locus coeruleus but may also be found in a related condition ( LEWY BODY DISEASE, DIFFUSE) cha racterized by dementia in combination with varying degrees of parkinsonism. (Adams et al., Principles of Neurology, 6th ed, p1059, pp1067-75) [MeSH D010300]. | |
| Cerebrovascular Disorders (A502) | A broad category of disorders characterized by impairment of blood flow in the arteries and veins which supply the brain. These include CEREBRAL INFARCTION; BRAIN ISCHEMIA; HYPOXIA, BRAIN; INTRACRANIAL EMBOLISM AND THROMBOSIS; INTRACRANIAL ARTERIOVENOUS MALFORMATIONS; and VASCULITIS, CENTRAL NERVOUS SYSTEM. In common usage, the term cerebrovascular disorders is not limited to conditions that affect the cerebrum, but refers to vascular disorders of the entire brain including the DIENCEPHALON; BRAIN STEM; and CEREBELLUM [MeSH D002561] | |
| Eye diseases, visual impairments (A503) | - Eye diseases [Mesh D005128], - Vision disorders: Visual impairments limiting one or more of the basic functions of the eye: visual acuity, dark adaptation, colour vision, or peripheral vision. These may result from EYE DISEASES; OPTIC NERVE DISEASES; VISUAL PATHWAY diseases; OCCIPITAL LOBE diseases; OCULAR MOTILITY DISORDERS; and other conditions. Visual disability refers to inability of the individual to perform specific visual tasks, such as reading, writing, orientation, or travelling unaided. (From Newell, Ophthalmology: Principles and Concepts, 7th ed, p132) [MeSH D014786] | |
| Dementia, cognitive impairment (A504) | Dementia: An acquired organic mental disorder with loss of intellectual abilities of sufficient severity to interfere with social or occupational functioning. The dysfunction is multifaceted and involves memory, behaviour, personality, judgment, attention, spatial relations, language, abstract thought, and other executive functions. The intellectual decline is usually progressive, and initially spares the level of consciousness. [Dementia: MeSH D003704]  This category includes also less severe cognitive impairments affecting the ability to think, concentrate, formulate ideas, reason and remember. | |
| Depression symptoms (A505) | Depression: Depressive states usually of moderate intensity in contrast with major depression present in neurotic and psychotic disorders. [Depression: MeSH D003863]  Depressive disorder: An affective disorder manifested by either a dysphoric mood or loss of interest or pleasure in usual activities. The mood disturbance is prominent and relatively persistent. [MeSH D003866]  Dysthymic Disorder: Chronically depressed mood that occurs for most of the day more days than not for at least 2 years. The required minimum duration in children to make this diagnosis is 1 year. During periods of depressed mood, at least 2 of the following additional symptoms are present: poor appetite or overeating, insomnia or hypersomnia, low energy or fatigue, low self esteem, poor concentration or difficulty making decisions, and feelings of hopelessness. [MeSH D019263] | |
| Syncope (A506) | A transient loss of consciousness and postural tone caused by diminished blood flow to the brain (i.e., BRAIN ISCHEMIA). Presyncope refers to the sensation of lightheadedness and loss of strength that precedes a syncopal event or accompanies an incomplete syncope. (From Adams et al., Principles of Neurology, 6th ed, pp367-9) [MeSH D013575]. | |
| Gait and/or balance impairment (A507) | Gait is the way one locomotes or walks [MeSH D005684]. Examples: walking patterns and running patterns; impairments such as spastic gait, hemiplegic gait, paraplegic gait, asymmetric gait, limping and stiff gait pattern [ICF b770].  Postural balance or musculoskeletal equilibrium: A state of the body being evenly balanced in [POSTURE](http://www.nlm.nih.gov/cgi/mesh/2007/MB_cgi?mode=&term=POSTURE). The biomechanical responses of the [MUSCULOSKELETAL SYSTEM](http://www.nlm.nih.gov/cgi/mesh/2007/MB_cgi?mode=&term=MUSCULOSKELETAL+SYSTEM) during standing, walking, sitting, and other movements [MeSH D004856].  Balance impairments include impairments of sitting, static standing or dynamic balance. In the context of falls gait and balance impairments are often detected with timed or qualitative performance tests such as the get up and go test. | |
| Urinary incontinence (A508) | Involuntary loss of [URINE](http://www.nlm.nih.gov/cgi/mesh/2007/MB_cgi?mode=&term=URINE), such as leaking of urine. It is a symptom of various underlying pathological processes. Major types of incontinence include [URINARY URGE INCONTINENCE](http://www.nlm.nih.gov/cgi/mesh/2007/MB_cgi?mode=&term=URINARY+URGE+INCONTINENCE) and [URINARY STRESS INCONTINENCE](http://www.nlm.nih.gov/cgi/mesh/2007/MB_cgi?mode=&term=URINARY+STRESS+INCONTINENCE) [MeSH D014549]. | |
| Screening tool (A509) | A fall screening tool is a short test intended to determine an older person’s risk of falling in order to determine eligibility for a fall risk intervention. It is not usually used to determine treatment received. Examples are the FRAT and AGS/BGS fall screening algorithm | |
| Others (A599/A599A) | Not described under A500-A509.  A599A: Brief description (free text) | |
| Medication specific (A600) | In which individuals have been selected as they are taking specified classes of medication with a known association with fall risk (e.g. SSRIs; sedatives; hypnotics) or as identified by the authors of the paper. | |
| Specific groups excluded | | |
| Dementia, cognitive impairment (A700) | Dementia: An acquired organic mental disorder with loss of intellectual abilities of sufficient severity to interfere with social or occupational functioning. The dysfunction is multifaceted and involves memory, behaviour, personality, judgment, attention, spatial relations, language, abstract thought, and other executive functions. The intellectual decline is usually progressive, and initially spares the level of consciousness. [Dementia: MeSH D003704]  This category includes also less severe cognitive impairments affecting the ability to think, concentrate, formulate ideas, reason and remember. | |
| Other specified exclusion (A799/A799A) | Specific group(s) stated by authors and which are not codable elsewhere.  Don’t code “Other specified exclusion” if the criteria interferes obviously with the planned intervention or increases the risk of dropping out of the study in an obvious way:  *Illustrative examples:*   - Terminal illness - Admitted for palliative care (institutional studies) - Enrolled in any other similar studies - Participating in any similar interventions - Receiving home nursing care on a regular basis (community studies) - Planning to be absent from the intervention location for a longer period or don’t expect to remain in the area during the intervention period - Psychiatric illness prohibiting participation - Too frail to withstand the exercises - Contraindication to treatment - Not speaking the language the intervention or assessment is delivered in - Living to far away from the research centre - Could not give informed consent (e.g.: cognitive impairment and no regular carer) - Unable or unwilling to complete the baseline assessments - Not ambulatory with or without an assistive device - Uncontrolled cardiac failure of hypertension - Chronic alcoholism - Active cardiovascular, pulmonary, vestibular and bone diseases - Active metabolic diseases   A799A: Brief description (free text) | |
| ***No selection criteria specified (A002)*** | Authors have not specified inclusion and exclusion criteria. | |

# Domain 2: Base

| Recruitment Site/Main Site (s) of Delivery | |
| --- | --- |
| This section classifies the recruitment site at which cases were identified, and the site at which the majority of the assessment and intervention are delivered. It is very possible that recruitment occurs at one site, and the delivery of services may occur across multiple sites.  For pharmacological studies the site of delivery is the site where the drugs are taken (e.g. if in community studies drugs are taken by the participants themselves code *participant’s home (B230), if drugs are taken directly under supervision of a community nurse code “providers of ambulant health care” (B220))*  Post intervention follow-up or booster sessions/visits are not counted in this category.  For the classification of the health care providers the International Classification for Health Accounts (ICHA) developed by the Organisation for Economic Co-operation and Development (OECD) was used as far as possible. | |
| Hospitals/Departments/Wards (Inpatient: Acute or Sub-acute) | |
| Acute (B100/B200) | Licensed establishments primarily engaged in providing diagnostic and medical treatment (both surgical and non-surgical) to in-patients with a wide variety of medical conditions.  Illustrative examples:   - General acute care hospitals; - Community, county, and regional hospitals; - Hospitals of private non-profit-organisations (e.g. Red Cross); - Teaching hospitals; university hospitals; - Army, veterans, and police hospitals; - Acute department of a geriatric hospital - Geriatric evaluation and treatment unit - Overnight acute inpatient services; - Some day acute inpatient services;   Emergency departments are classified under B111/B211 |
| Emergency department (B101/B201) | Hospital department responsible for the administration and provision of immediate medical or surgical care to the emergency patient. |
| Sub-acute (B102/B202) | Licensed establishments or departments primarily engaged in medical post-acute, rehabilitative, preventive and extended care services   - Geriatric rehabilitation (inpatient) - Rehabilitation hospital - Long-stay geriatric care in the acute hospital setting |
| Nursing and residential care facilities (Non-acute) [ICHA-HP HP.2] | |
| Nursing care facilities & Community care facilities for the elderly(B110/B210) | **Nursing care facilities:** Establishments primarily engaged in providing in-patient nursing and rehabilitative services. The care is generally provided for an extended period of time to individuals requiring nursing care. These establishments have a permanent core staff of registered or licensed practical nurses who, along with other staff, provide nursing and continuous personal care services. *Note:* medical nursing care facilities provide predominantly long-term care but also occasionally acute health care and nursing care in conjunction with accommodation and other types of social support such as assistance with day-to-day living tasks and assistance towards independent living. Nursing homes provide long-term care involving regular basic nursing care to chronically ill, frail, disabled or convalescent persons or senile persons placed in an in-patient institution. Health care and treatment have to constitute an important part of the activities provided to be included in the SHA. Hostels with only limited medical assistance, such as supervision of compliance with medication, should be excluded. [ICHA-HP.2.1]  Illustrative examples   - Convalescent homes or convalescent hospitals - Homes for the elderly with nursing care; - In-patient care hospices; - (Community) nursing homes; - Rest homes with nursing care; - Skilled nursing facilities (USA); - Teaching nursing homes; - Long-term care facilities   **Community care facilities for the elderly:** Establishments primarily engaged in providing residential and personal care services for elderly and other persons (1) unable to fully care for themselves and/or (2) unwilling to live independently. The care typically includes room, board, supervision, and assistance in daily living, such as housekeeping services. In some instances these establishments provide skilled nursing care for residents in separate on-site facilities. Assisted living facilities with on-site nursing care facilities are included in this item. Homes for the elderly without on-site nursing care facilities are also included. [ICHA-HP.2.3]  Illustrative examples   - Assisted-living facilities; - Residential (rest) homes; - Continuing-care retirement communities; - Homes/apartments for the elderly without nursing care. |
| Providers of ambulatory health care [ICHA-HP HP.3] | |
| **Recruitment:**  If the recruitment procedure was based on e.g. computerized patient registers from general practitioners code “community based”. Code “Providers of ambulatory health care” only if they have an active role in screening and recruiting eligible study participants inside their practices or services.  **Delivery:**  If drugs are described by providers of ambulatory health care code this category. | |
| Offices of physicians & Offices of other health practitioners & Out-patient care centres/departments/wards & Providers of home health care services & Other providers of ambulatory health care (B120/B220) | **Offices of physicians:** Establishments of health practitioners holding the degree of a doctor of medicine or a qualification at a corresponding level (ISCO-88 fourth degree level), primarily engaged in the independent practice of general or specialised medicine or surgery. These practitioners operate private or group practices in their own offices or in the facilities of others, such as hospitals or health maintenance organizations (HMO) type medical centres. [ICHA-HP.3.1]  Illustrative examples   - General practitioners in private offices; general practices - Specialists of a wide range of specialities in private offices; - Establishments known as medical clinics which are primarily engaged in the treatment of outpatients (Korea, Japan).   **Offices of other health practitioners:** Establishments of independent health practitioners (other than physicians), such as chiropractors, optometrists, mental health specialists, physical, occupational, and speech therapists and audiologists establishments primarily engaged in providing care to outpatients. These practitioners operate private or group practices in their own offices (*e.g*., centres, clinics) or in the facilities of others, such as hospitals or HMO medical centres. *Note:* this item includes paramedical practitioners providing so-called “traditional medicine” without a doctor’s approbation. Some form of legal registration and licensing (implying a minimum of public control over the contents of care provided) is regarded as a necessary condition in order to be reported as paramedical practitioner in many countries. [ICHA-HP.3.3]  Illustrative examples   - Nurses; - Physiotherapists and physical therapists; - Occupational and speech therapists; - Audiologists; - Dieticians’ offices; - Podiatrists’ offices; - Registered or licensed practical nurses’ offices; - Forms of traditional medicine; formal licensing may not be required as criteria for recognition as health practitioner in countries where these forms of medicine have been an integral part of medical practice for a long time; - Oriental (traditional) medicine clinics (Korea).   **Out-patient care centres/departments/wards:** Establishments engaged in providing a wide range of out-patient services by a team of medical, paramedical and often also support staff, usually bringing together several specialities and/or serving specific functions of primary care. These establishments generally treat patients who do not require in-patient treatment. [ICHA-HP.3.4]  This category includes   - Free-standing ambulatory surgery centres (This item comprises establishments with physicians and other medical staff primarily engaged in providing surgical services (*e.g*., orthoscopic and cataract surgery) on an out-patient basis. Out-patient surgical establishments have specialised facilities, such as operating and recovery rooms, and specialised equipment, such as anaesthetic or X-ray equipment.) - All other out-patient multi-speciality and co-operative services centres. Establishments with medical staff primarily engaged in providing general or specialised out-patient care. Centres or clinics of health practitioners with different degrees from more than one speciality practising within the same establishment are included in this item. *Note*: included are health maintenance organisation (HMO) medical centres and clinics. HMO type medical centres comprise establishments with physicians and other medical staff primarily engaged in providing a range of outpatient health care services to the HMO subscribers with a focus generally on primary health care. These establishments are owned by the HMO. Included are HMO establishments that both provide health care services and underwrite health and medical insurance policies. Included are integrated community care centres providing both in-patient and out-patient services primarily engaged in out-patient services.   Illustrative examples   - Out-patient community centres and clinics; - Multi-speciality out-patient polyclinics; - Multi-speciality HMO medical centres and clinics. - HMO research centres - Day-hospitals/clinics - Out-patient department - Out-patient geriatric rehabilitation - Day-wards - Ambulatory care centres   **Providers of home health care services:** Establishments primarily engaged in providing skilled nursing services in the home, along with a range of the following: personal care services; homemaker and companion services; physical therapy; medical social services; medications; medical equipment and supplies; counselling; 24-hour home care; occupation and vocational therapy; dietary and nutritional services; speech therapy; audiology; and high-tech care, such as intravenous therapy. [ICHA-HP.3.6]  Illustrative examples   - community nurses and domiciliary nursing care; - home health care agencies; visiting nurse associations.   **Other providers of ambulatory health:** Establishments primarily engaged in providing ambulatory health care services (other than offices of physicians, dentists, and other health practitioners; out-patient care centres; medical laboratories and diagnostic imaging centres; and home health care providers). [ICHA-HP.3.9]  *Providers of all other ambulatory health care services*  Establishments primarily engaged in providing ambulatory health care services (other than offices of physicians, dentists, and other health practitioners; out-patient care centres; home health care providers).  Illustrative examples   - health screening services (except by offices of health practitioners); - hearing testing services (except by offices of audiologists); - pacemaker monitoring services; - physical fitness evaluation services (except by offices of health practitioners); - research centres targeting health issues (e.g. nutrition research centre) |
| Community based recruitment/delivery **Please consider that the “main site of delivery” for pharmacological studies is the site where the drugs are prescribed.** | |
| Community based (B130) | - Public advertising/ presentations - Untargeted public advertising and presentation in the community - Press - Radio - Advertising campaign - Organisation/institution related recruitment - Senior centres - Community centres - Clubs - Churches - Registers of health care recipients (e.g Health Maintenance Organisations, General Practice lists, Primary care providers) - Ex-patients of an health care provider (e.g., addresses form registers of the accident and emergency department and orthopaedic fracture clinic) - Directory of veterans’ organizations - HMO members - Social security funds - Social health insurance and sickness funds - Social health insurance covering various groups of state employees (army, veterans, railroad and other public transport, police, state officials, etc.) - Private insurances - Participants of previous studies |
| Population based registers (B140) | Using the addresses of public registers for recruiting participants. (e.g., via mailing or telephone)  Illustrative examples   - Voter registration lists - Electoral roll   [If for recruitment community based and population based strategies were used code only B130 (Community based)] |
| Participants home (B230) | Interventions or procedures delivered in participants home (including indoor, entrance and private outdoor) |
| Organisations & other locations in the community (B231) | - Senior centres - Community centres - Clubs - Churches   Not described under hospitals, nursing and residential care facilities and other provider of ambulatory health care. |
| Outdoor environment (B240) | Public outdoor environment. |
| Others (B199/B299) & (B199A/B299A) | Others not described under B100-B140  B199A & B299A: Brief description (free text) |
| Not described B100/B200) | Source of case identification is not or incompletely described |
| Assessments delivered by | |
| Professionals (B300) | Assessments delivered by any kind of health care professional. |
| Trained non-professionals (B301) | Formal or informal non-professionals trained by professional instructors in specific assessments. |
| Self-Assessment (B302) | Self assessment done by the participant according standardised instruction or material (e.g. paper self assessment). |
| Others (B399/B399A) | It is not or incompletely described who delivers the assessments.  B399A: Brief description (free text) |
| Interventions & postintervention follow-ups delivered by | |
| For pharmacological studies please code the profession that is prescribing the drugs. | |
| Professionals | |
| The classification of health care professionals is largely based on the International Standard Classification of Occupants (ISCO-88). | |
| Medical doctors/ Medical assistants (B400) | Medical doctors conduct research, improve or develop concepts, theories and operational methods, and apply preventive or curative measures. (ISCO-88:2221)  *Illustrative examples*   - Physician - Geriatricians - Clinicians - Physical medicine and rehabilitation doctor   Medical assistants carry out advisory, diagnostic, preventive and curative medical Tasks, more limited in scope and complexity than those carried out by Medical doctors. They work independently or with the guidance and supervision of Medical doctors in institutions or in the field as part of the public health service, and may work mainly with diseases and disorders common in their region, or mainly apply specific types of treatment. (ISCO-88:3221)  *Illustrative examples*   - Physician’s assistant |
| Pharmacists (B401) | Pharmacists apply pharmaceutical concepts and theories by preparing and dispensing or selling medicaments and drugs. (ISCO-88:2224) |
| Nursing (associate) professionals (B402) | Nursing professionals assist medical doctors in their tasks, deal with emergencies in their absence, and provide professional nursing care for the sick, injured, physically and mentally disabled, and others in need of such care (…).(ISCO-88:2230 & 3231)  *Illustrative examples*   - Study nurse - Research nurse - Trained nurses - Nurses - Trained district nurse - Nurse practitioner - Community nurse - Public health nurses |
| Social work (associate) professionals (B403) | Social work professionals provide guidance to clients in social and related matters to enable them to find and use resources to overcome difficulties and achieve particular goals. (ISCO-88:2446 & 3460)  *Illustrative examples*   - Welfare worker - Social worker |
| Psychologists (B404) | Psychologists research into and study mental processes and behaviour of human beings as individuals or in groups, and apply this knowledge to promote personal, social, educational or occupational adjustment and development. (ISCO-88:2445) |
| Physiotherapists & related associate professionals (B405) | Physiotherapists and related associate professionals treat disorders of bones, muscles and parts of the circulatory or the nervous system by manipulative methods, and ultrasound, heating, laser or similar techniques, or apply physiotherapy and related therapies as part of the treatment for the physically disabled, mentally ill or unbalanced. (ISCO-88:3226)  *Illustrative examples*   - Exercise instructors - Instructors - Physiotherapist - Physical therapist - Physical research therapist - Trained exercise physiologists - Professional instructor - Physiology graduate students |
| Occupational therapists (B406) | Occupational therapists assess, plan, organize, and participate in rehabilitative programs that help restore vocational, homemaking, and daily living skills, as well as general independence, to disabled persons. |
| Modern health associate professionals (except nursing) not elsewhere classified (B407) | This unit group covers modern health associate professionals (except nursing) not classified elsewhere. (ISCO-88:3229)  *Illustrative examples*   - Health behaviourist |
| Unspecified multidisciplinary/research teams (B408) | *Illustrative examples*   - Staff-members - Research staff |
| Other professionals  (B499/B499A) | Other professionals not described under D400-D408.  B499A: Brief description (free text) |
| Trained non-professionals | |
| Formal (B500) | Trained non-professionals with a contract of labour and payment or students within the scope of their studies. |
| Informal (B501) | *Illustrative examples*   - Voluntary workers - Family members or caregivers |
| Other non-professional (B599/B599A) | Other non-professional not described under B500-& B501.  B599A: Brief description (free text) |
| Self management interventions (B600) | Self-management interventions are intended to help people understand how individual behaviours can affect how much an illness interferes with their lives and to act on the basis of that understanding. Self-management programs addresses e.g. areas like disease and health management, role management and emotional management. The techniques being used includes e.g. self-monitoring, self-evaluation, self-instruction, goal-setting and strategy instruction.  Self-help interventions can involve professionals, but they usually include only limited direct contact with a professional. (Code the profession that delivers the self-management techniques as well). |
| Institutions/  authorities (B700) | Particularly environmental interventions (e.g. new pavements, new floor covering in a nursing home) or health education campaigns that are targeting population or population groups without any specific personal interaction that could be better described by the categories B400-B600. |
| ***Other*** | |
| Others (B999/B999A) | Others not described under B400-B700  B999A: Brief description (free text) |
| Not described (B004) | It is not or incompletely described who delivers the intervention. |

# Domain 3: Components

| Components | |
| --- | --- |
| Notes:  This section describes components of the intervention. This part includes the assessments and the kind of combination of the interventions described in domain 4. Please consider that only assessments that are part of the intervention (as opposed to research study baseline or follow up assessments in published studies) should be detailed.  Possible subsequent interventions or action after assessment based referrals or recommendations that are not described and controlled by the study protocol are included in the coding of this section. | |
| Assessment as part of the intervention (generic) | |
| CGA (generic) (C100) | The goals of comprehensive geriatric assessment are: (1) to improve diagnostic accuracy, (2) to guide the selection of interventions to restore or preserve health, (3) to recommend an optimal environment for care, (4) to predict outcomes, and (5) to monitor clinical change over time.  In addition to the patient, the process often includes family members and other important persons in the individual's environment. It is conducted by a core team that consists, at a minimum, of a physician, nurse, and social worker, each with special expertise in caring for older people. Frequently, a psychiatrist is a member of the core team.  This interdisciplinary diagnostic process intended to determine an older person's physical and mental health, social and economical status, functional status, environmental characteristic in order to develop and implement a care plan. *(Source: National Institutes of Health. Consensus Development Conference Statement. October 19-21, 1987)* |
| Assessment as part of the intervention (specific) | |
| Fall risk assessment (C200) | Fall risk assessment is a diagnostic process intended to determine an older person’s risk of falling in order to plan coordinated treatment and long-term follow up. The fall risk assessment is sometimes performed in specialized settings like a fall clinic. The assessment includes methods that are specifically designed and tested for the assessment of the risk of falling (e.g. gait speed, static balance, strength, dual task measures, cardio-vascular assessments etc).  e.g. PPA (Physiological Profile Assessment) |
| Gait and balance (only) (C201) | Assessments that determine the quality of gait and/or balance. |
| Cardiovascular assessment (C202) | Assessment of basic cardiovascular status including heart rate and rhythm, postural pulse and blood pressure and, if appropriate, heart rate and blood pressure responses to carotid sinus stimulation. *(Source: AGS Panel of Falls Prevention, JAGS 2001)* |
| Medication review (C203) | Performing a comprehensive medication review to identify, resolve, and prevent medication-related problems, including adverse drug events with a special focus on medications associated with an increased fall risk (e.g.  antipsychotics, sedatives, hypnotics, antidepressants, antiarrhythmics, anticonvulsants, anxiolytics, antihypertensives, diuretics). |
| Vision (C204) | Vision assessment (e.g. visual acuity, depth perception, contrast sensitivity, cataracts) |
| Foot assessment (C205) | Assessment of the foot, usually undertaken by a podiatrist or chiropodist. May include assessment of biomechanical alignment; pain; callus; footwear. |
| Psychological assessment (C206) | Psychological assessments in this area focuses particularly self efficacy and confidence or fear of falling with specific instruments (e.g. FES-I ). Further domains are e.g. behavioural problems and risk behaviours. |
| Environment (dwelling units) (C207) | Includes formal home visit assessments schedules e.g. Housing Enabler, |
| Environment (public) (C208) | Assessment of the hazards, safety and/or enabling features of the external environment such as footpath assessment. |
| Environment (aids for personal care and protection) (C209) | Assessment to determine the need for aids for personal care, including mobility aids, dressing aids. Protective aids include hip protectors and alarm systems. [Footwear assessment as part of a podiatric assessment code C205 (Foot assessment)]. |
| Others (C299/C299A) | Other assessments not described under C200-C209.  C299A: Brief description (free text) |
|  |  |
| Combination | |
| Most interventions fall under the following sub-domains (detailed under Domain 4: Descriptors of the intervention).   - Exercises (supervised and/or unsupervised) - Medication (drug target) - Surgery - Management of urinary incontinence - Fluid or nutrition therapy - Psychological - Environment/Assistive technology - Social environment - Other interventions/procedures   (Knowledge is not counted as a separate category)  Combination refers to how many sub-domains are delivered to the participants of an intervention, and importantly, the manner in which these sub-domains are combined. | |
| Single (C300) | Only one major sub-domain of intervention is provided to the participants. Different interventions of one sub-domain are counted as single intervention as well. If an intervention (e.g. furnishing and adaptations to homes) is based on a specific assessment (e.g. environment / dwelling units) count this intervention also as a single intervention. If only one or more assessments were delivered in the same setting without any interventions in terms of specific action count detailed in the article code also only as single intervention.  *Illustrative examples:*   - Supervised exercises.   *If all participants only received supervised exercise, this would be a single intervention. It maybe possible to describe further the type of supervised exercise, eg strength/resistance and general physical activity. Even if patients received a different mix of supervised exercise types this would be a single intervention as it all falls under the same sub-domain.*   - Home hazard management   *If all participants in an intervention group received a home visit to assess and modify environmental hazards, this would be a single intervention type.*   - Foot assessment - Cardiovascular assessment |
| Multiple (C301) | Interventions in which two or more sub-domains of intervention are given to every participant of the falls prevention programme. Combinations of interventions (e.g. vitamin D) and an assessment of another domain (environment / dwelling units) code as multiple interventions.  *Illustrative examples:*  All participants of the fall prevention programme receive   - Medication (drug target) + supervised exercise - Supervised exercise + staff training - Geriatric assessment + environmental assessment in the patient’s home |
| Multifactorial (C302) | Interventions in which two or more sub-domains of intervention can be given to participants, but the interventions are linked to each individual’s risk profile (usually assessed using a formal process such as the PPA). Unlike multiple interventions, not all participants in a programme receive the same combination of sub-domains.  *Illustrative examples:*   - Each individual receives an assessment of known risk factors for falling (fall risk assessment) and receives an intervention matched to their risk factor profile. - Participation in any possible combination of intervention options according the participant’s choice |

# Domain 4: Descriptors

| Descriptors | |
| --- | --- |
| Interventions should be coded according to their intended mode of delivery as opposed to actual mode of delivery. | |
| Exercises (supervised/unsupervised) | |
| **Supervised exercises** | Exercise sessions supervised by a professional, trained non-professional or volunteer. Supervised exercises require a personal direct contact with the instructor. |
| **Unsupervised Exercises** | Recommended exercises without supervision. |
| **Duration (months) (D10A/D1AA)** | Supervised exercises: The period the individual training or the training class is offered to the participant.  Unsupervised exercises: The recommended period for doing the exercises.. 99=not described |
| **Frequency (per month) (D10B/D1AB))** | Supervised exercises: The frequency the individual training or the training class is offered to the participant.  Unsupervised exercises: The recommended frequency for doing the exercises.  99=not described |
| **Intensity (D10C/D1AC)** | Can be a subjective or objective assessment. Intensity can be assessed in a number of different ways. [1] Self-perceived exertion (e.g Borg Self-perceived exertion scale). [2] In relation to the One Repetition Maximum ( the maximum amount of weight that can be lifted by a muscle) [3] Expressed as a % of a measured cardiovascular index of intensity (eg heart rate) [4] Physiological responses such as breathlessness or increased heart rate. [5] In relation to the limits of stability for balance exercises, and decreasing base of support. Authors of reports should report the desired intensity of the exercise programme in relation to one of these recognised methods or a validated alternative (consult ACSM guidelines for exercise prescription and testing).  1=low, 2=moderate, 3=hard 99=not described |
| **Individual/Group (D10D/D1AD)** | Describes whether the exercise intervention is delivered on a one to one basis, or are part of a group of individuals.  1=individual, 2=group, 3=combination, 99=not described |
| Gait, balance, and functional training (D100/D1A0) | *Gait training* involves specific correction of walking technique (e.g., posture, stride length and cadence) and changes of pace, level and direction. *Balance Training* involves the efficient transfer of bodyweight from one part of the body to another or challenges specific aspects of the balance systems (eg vestibular systems). Balance retraining activities range from the re-education of basic functional movement patterns to a wide variety of dynamic activities that target more sophisticated aspects of balance. *Functional training* utilises functional activities as the training stimulus, and is based on the theoretical concept of task specificity. All gait, balance and functional training should be based on an *assessment* of the participant’s abilities prior to starting the program; *tailoring* of the intervention to the individuals abilities; and *progression* of the exercise program as ability improves.  *Illustrative examples:*   - Gait training - Heel raises, toe raises, walking on the toes/ heels, heel to toe walking, walking backward, forwards, sideways, turning, bending, stepping (stair climbing where available) side stepping. - Vestibular and proprioceptive retraining exercises (e.g. Cawthorne Cooksey exercises, different head and eye positions etc) - Specific exercises involving changes in pace, level, head and eye gaze, obstacle courses etc. - Ball exercises (and other co-ordination and reaction activities) - Foot eye coordination - Psychomotor performance (reaction time) - Obstacle courses - Standing on unstable surface - Walking in line - Reactive games - Dynamic balance exercises include knee bends (squats), calf raises and toe raises - Static balance exercises include standing in one leg or tandem standing - Walking exercises include turning, tandem, backward and sideways walking |
| Strength/resistance (incl. power) (D101/D1A1) | The term Resistance Training covers all types of weight training ie contracting the muscles against a resistance to ‘overload’ and bring about a training effect in the muscular system. The resistance is an external force, which can be ones own body placed in an unusual relationship to gravity (e.g. prone back extension) or an external resistance (e.g. free weight). All strength/resistance training should be based on an *assessment* of the participant’s abilities prior to starting the program; *tailoring* of the intervention to the individuals abilities; and *progression* of the exercise program as ability improves.  *Illustrative examples:*   - weight training (free weights, fixed resistance equipment, resistance/ theraband bands and also body weight in the early stages of training) - Functional training with added weight (ie. weighted shopping bags etc) - Propulsion (explosive movement) training for power (eg. jumping) - Pilates resistance exercises - Weight lifting with free weights - Exercise on machines - Cable pulleys |
| Flexibility (D102/D1A2) | Flexibility Training is the planned process by which stretching exercises are practised and progressed to restore or maintain the optimal Range Of Movement (ROM) available to a joint or joints. The ranges of motion used by flexibility programs may vary from restoration/maintenance of the entire physiological range of motion, or alternatively, maintenance of range that is essential to mobility or other functions.  *Illustrative examples:*   - Static Stretches (eg. hamstring/calf/chest/side stretch) - Pilates flexibility training (eg static and moving stretches) - Yoga |
| 3 D (D103/D1A3) | 3D training involves constant movement in a controlled, fluid, repetitive way through all 3 spatial planes or dimensions (forward and back, side to side, and up and down). Tai Chi and Qi Gong incorporate specific weight transferences and require upright posture and subtle changes of head position and gaze direction. Dance involves a wide range of dynamic movement qualities, speeds and patterns.  *Illustrative examples:*   - Tai Chi - Qi Gong - Dance |
| General physical activity (D104/D1A4) | *Physical Activity* is any bodily movement produced by skeletal muscle contraction resulting in a substantial increase in energy expenditure. Physical activity has both an occupational, transporational and recreational components and includes pursuits like golf, tennis, and swimming. It also includes other active pastimes like gardening, cutting wood, and carpentry. Physical activity can provide progressive health benefits and be a catalyst for improving health attitudes, health habits, and lifestyle. Increasing habitual physical activity should be with specific recommendations as to duration, frequency and intensity if a physical or mental health improvement is indicated.  *Illustrative examples:*   - Walking indoors and outdoors - Swimming and cycling. |
| Endurance (D105/D1A5) | *Endurance training* is aimed at cardiovascular conditioning and is aerobic in nature and simultaneously increases the heart rate and the return of blood to the heart.  *Illustrative examples:*   - Walking (30 mins 5 x p/w at a moderate/brisk pace) - Cycle Ergometer - Treadmill walking - Rowing machines - Continuous marching etc. during exercise class - Brisk-walking - Jogging - Interval training/speed-play/Fartlek training |
| Others (D109/D1A9)  & (D109A/D1A9A) | Other kind of exercises not described under D100-D105/D1A0-D1A5.  D109A/D1A9A: Brief description (free text) |
| Medication (Drug Target) | |
| The categories of drugs are based upon the Anatomical Therapeutic Chemical (ATC). Code this item if the intervention includes direct action targeted to specific classes of drugs (withdrawal, dose reduction or increase, substitution, provision) according to protocol and reported in the article. Medication reviews with recommendations for the physician of the participant code under assessment. | |
| Antihypertensives (D200) | **Antihypertensives [C02]**   - C02A [ANTIADRENERGIC AGENTS, CENTRALLY ACTING](http://www.whocc.no/atcddd/indexdatabase/index.php?query=C02A) - C02B [ANTIADRENERGIC AGENTS, GANGLION-BLOCKING](http://www.whocc.no/atcddd/indexdatabase/index.php?query=C02B) - C02C [ANTIADRENERGIC AGENTS, PERIPHERALLY ACTING](http://www.whocc.no/atcddd/indexdatabase/index.php?query=C02C) - C02D [ARTERIOLAR SMOOTH MUSCLE, AGENTS ACTING ON](http://www.whocc.no/atcddd/indexdatabase/index.php?query=C02D) - C02K [OTHER ANTIHYPERTENSIVES](http://www.whocc.no/atcddd/indexdatabase/index.php?query=C02K) - C02L [ANTIHYPERTENSIVES AND DIURETICS IN COMBINATION](http://www.whocc.no/atcddd/indexdatabase/index.php?query=C02L) - C02N [COMBINATIONS OF ANTIHYPERTENSIVES IN ATC-GR.](http://www.whocc.no/atcddd/indexdatabase/index.php?query=C02N) |
| Other cardiovascular agents (D201) | **Cardiovascular System [C] without Antihypertensives**   - C01 [CARDIAC THERAPY](http://www.whocc.no/atcddd/indexdatabase/index.php?query=C01) - C03 [DIURETICS](http://www.whocc.no/atcddd/indexdatabase/index.php?query=C03) - C04 [PERIPHERAL VASODILATORS](http://www.whocc.no/atcddd/indexdatabase/index.php?query=C04) - C05 [VASOPROTECTIVES](http://www.whocc.no/atcddd/indexdatabase/index.php?query=C05) - C07 [BETA BLOCKING AGENTS](http://www.whocc.no/atcddd/indexdatabase/index.php?query=C07) - C08 [CALCIUM CHANNEL BLOCKERS](http://www.whocc.no/atcddd/indexdatabase/index.php?query=C08) - C09 [AGENTS ACTING ON THE RENIN-ANGIOTENSIN SYSTEM](http://www.whocc.no/atcddd/indexdatabase/index.php?query=C09) - C10 [LIPID MODIFYING AGENTS](http://www.whocc.no/atcddd/indexdatabase/index.php?query=C10) |
| Vitamin D (D202) | [**Vitamin D and analogues**](http://www.whocc.no/atcddd/indexdatabase/index.php?query=A11CC) **[A11CC]**   - A11CC01 [Ergocalciferol](http://www.whocc.no/atcddd/indexdatabase/index.php?query=A11CC01) - A11CC02 [Dihydrotachysterol](http://www.whocc.no/atcddd/indexdatabase/index.php?query=A11CC02) - A11CC03 [Alfacalcidol](http://www.whocc.no/atcddd/indexdatabase/index.php?query=A11CC03) - A11CC03 [Alfacalcidol](http://www.whocc.no/atcddd/indexdatabase/index.php?query=A11CC03) - A11CC04 [Calcitriol](http://www.whocc.no/atcddd/indexdatabase/index.php?query=A11CC04) - A11CC04 [Calcitriol](http://www.whocc.no/atcddd/indexdatabase/index.php?query=A11CC04) - A11CC05 [Colecalciferol](http://www.whocc.no/atcddd/indexdatabase/index.php?query=A11CC05) - A11CC06 [Calcifediol](http://www.whocc.no/atcddd/indexdatabase/index.php?query=A11CC06) - A11CC07 [Paricalcitol](http://www.whocc.no/atcddd/indexdatabase/index.php?query=A11CC07) - A11CC20 [Combinations](http://www.whocc.no/atcddd/indexdatabase/index.php?query=A11CC20) |
| Calcium (D203) | [**Calcium**](http://www.whocc.no/atcddd/indexdatabase/index.php?query=A12AA) **[A12AA]**   - A12AA01 [Calcium phosphate](http://www.whocc.no/atcddd/indexdatabase/index.php?query=A12AA01) - A12AA02 [Calcium glubionate](http://www.whocc.no/atcddd/indexdatabase/index.php?query=A12AA02) - A12AA03 [Calcium gluconate](http://www.whocc.no/atcddd/indexdatabase/index.php?query=A12AA03) - A12AA03 [Calcium gluconate](http://www.whocc.no/atcddd/indexdatabase/index.php?query=A12AA03) - A12AA04 [Calcium carbonate](http://www.whocc.no/atcddd/indexdatabase/index.php?query=A12AA04) - A12AA05 [Calcium lactate](http://www.whocc.no/atcddd/indexdatabase/index.php?query=A12AA05) - A12AA06 [Calcium lactate gluconate](http://www.whocc.no/atcddd/indexdatabase/index.php?query=A12AA06) - A12AA07 [Calcium chloride](http://www.whocc.no/atcddd/indexdatabase/index.php?query=A12AA07) - A12AA08 [Calcium glycerylphosphate](http://www.whocc.no/atcddd/indexdatabase/index.php?query=A12AA08) - A12AA09 [Calcium citrate lysine complex](http://www.whocc.no/atcddd/indexdatabase/index.php?query=A12AA09) - A12AA10 [Calcium glucoheptonate](http://www.whocc.no/atcddd/indexdatabase/index.php?query=A12AA10) - A12AA11 [Calcium pangamate](http://www.whocc.no/atcddd/indexdatabase/index.php?query=A12AA11) - A12AA12 [Calcium acetate anhydrous](http://www.whocc.no/atcddd/indexdatabase/index.php?query=A12AA12) - A12AA20 [Calcium (different salts in combination)](http://www.whocc.no/atcddd/indexdatabase/index.php?query=A12AA20) - A12AA30 [Calcium laevulate](http://www.whocc.no/atcddd/indexdatabase/index.php?query=A12AA30) |
| Other bone health medication (D204) | [**DRUGS AFFECTING BONE STRUCTURE AND MINERALIZATION**](http://www.whocc.no/atcddd/indexdatabase/index.php?query=M05B) **[M05B]**   - M05BA [Bisphosphonates](http://www.whocc.no/atcddd/indexdatabase/index.php?query=M05BA) - M05BB [Bisphosphonates, combinations](http://www.whocc.no/atcddd/indexdatabase/index.php?query=M05BB) - M05BC [Bone morphogenetic proteins](http://www.whocc.no/atcddd/indexdatabase/index.php?query=M05BC) - M05BX [Other drugs affecting bone structure and mineralization](http://www.whocc.no/atcddd/indexdatabase/index.php?query=M05BX) |
| Drugs used in diabetes (D205) | [**DRUGS USED IN DIABETES**](http://www.whocc.no/atcddd/indexdatabase/index.php?query=A10) **[A10]**   - A10A [INSULINS AND ANALOGUES](http://www.whocc.no/atcddd/indexdatabase/index.php?query=A10A) - A10B [BLOOD GLUCOSE LOWERING DRUGS, EXCL. INSULINS](http://www.whocc.no/atcddd/indexdatabase/index.php?query=A10B) - A10X [OTHER DRUGS USED IN DIABETES](http://www.whocc.no/atcddd/indexdatabase/index.php?query=A10X) |
| Anti-Parkinson drugs (D206) | [**ANTI-PARKINSON DRUGS**](http://www.whocc.no/atcddd/indexdatabase/index.php?query=N04) **[N04]**   - N04A [ANTICHOLINERGIC AGENTS](http://www.whocc.no/atcddd/indexdatabase/index.php?query=N04A) - N04B [DOPAMINERGIC AGENTS](http://www.whocc.no/atcddd/indexdatabase/index.php?query=N04B) |
| Anti-dementia drugs (D207) | [**ANTI-DEMENTIA DRUGS**](http://www.whocc.no/atcddd/indexdatabase/index.php?query=N06D) **[N06D]**   - N06DA [Anticholinesterases](http://www.whocc.no/atcddd/indexdatabase/index.php?query=N06DA) - N06DX [Other anti-dementia drugs](http://www.whocc.no/atcddd/indexdatabase/index.php?query=N06DX) |
| Antidepressants (D208) | [**ANTIDEPRESSANTS**](http://www.whocc.no/atcddd/indexdatabase/index.php?query=N06A) **[N06A]**   - N06AA [Non-selective monoamine reuptake inhibitors](http://www.whocc.no/atcddd/indexdatabase/index.php?query=N06AA) - N06AB [Selective serotonin reuptake inhibitors](http://www.whocc.no/atcddd/indexdatabase/index.php?query=N06AB) - N06AF [Monoamine oxidase inhibitors, non-selective](http://www.whocc.no/atcddd/indexdatabase/index.php?query=N06AF) - N06AG [Monoamine oxidase A inhibitors](http://www.whocc.no/atcddd/indexdatabase/index.php?query=N06AG) - N06AX [Other antidepressants](http://www.whocc.no/atcddd/indexdatabase/index.php?query=N06AX) |
| Antipsychotic/Neuroleptic drugs (D209) | [**ANTIPSYCHOTICS**](http://www.whocc.no/atcddd/indexdatabase/index.php?query=N05A) **[N05A]**   - N05AA [Phenothiazines with aliphatic side-chain](http://www.whocc.no/atcddd/indexdatabase/index.php?query=N05AA) - N05AB [Phenothiazines with piperazine structure](http://www.whocc.no/atcddd/indexdatabase/index.php?query=N05AB) - N05AC [Phenothiazines with piperidine structure](http://www.whocc.no/atcddd/indexdatabase/index.php?query=N05AC) - N05AD [Butyrophenone derivatives](http://www.whocc.no/atcddd/indexdatabase/index.php?query=N05AD) - N05AE [Indole derivatives](http://www.whocc.no/atcddd/indexdatabase/index.php?query=N05AE) - N05AF [Thioxanthene derivatives](http://www.whocc.no/atcddd/indexdatabase/index.php?query=N05AF) - N05AG [Diphenylbutylpiperidine derivatives](http://www.whocc.no/atcddd/indexdatabase/index.php?query=N05AG) - N05AH [Diazepines, oxazepines and thiazepines](http://www.whocc.no/atcddd/indexdatabase/index.php?query=N05AH) - N05AK [Neuroleptics, in tardive dyskinesia](http://www.whocc.no/atcddd/indexdatabase/index.php?query=N05AK) - N05AL [Benzamides](http://www.whocc.no/atcddd/indexdatabase/index.php?query=N05AL) - N05AN [Lithium](http://www.whocc.no/atcddd/indexdatabase/index.php?query=N05AN) - N05AX [Other antipsychotics](http://www.whocc.no/atcddd/indexdatabase/index.php?query=N05AX) |
| Anxyolitics, hypnotics & sedatives (D210) | [**ANXIOLYTICS**](http://www.whocc.no/atcddd/indexdatabase/index.php?query=N05B) **[N05B]**   - N05BA [Benzodiazepine derivatives](http://www.whocc.no/atcddd/indexdatabase/index.php?query=N05BA) - N05BB [Diphenylmethane derivatives](http://www.whocc.no/atcddd/indexdatabase/index.php?query=N05BB) - N05BC [Carbamates](http://www.whocc.no/atcddd/indexdatabase/index.php?query=N05BC) - N05BD [Dibenzo-bicyclo-octadiene derivatives](http://www.whocc.no/atcddd/indexdatabase/index.php?query=N05BD) - N05BE [Azaspirodecanedione derivatives](http://www.whocc.no/atcddd/indexdatabase/index.php?query=N05BE) - N05BX [Other anxiolytics](http://www.whocc.no/atcddd/indexdatabase/index.php?query=N05BX)   [**HYPNOTICS AND SEDATIVES**](http://www.whocc.no/atcddd/indexdatabase/index.php?query=N05C) **[N05C]**   - N05CA [Barbiturates, plain](http://www.whocc.no/atcddd/indexdatabase/index.php?query=N05CA) - N05CB [Barbiturates, combinations](http://www.whocc.no/atcddd/indexdatabase/index.php?query=N05CB) - N05CC [Aldehydes and derivatives](http://www.whocc.no/atcddd/indexdatabase/index.php?query=N05CC) - N05CD [Benzodiazepine derivatives](http://www.whocc.no/atcddd/indexdatabase/index.php?query=N05CD) - N05CE [Piperidinedione derivatives](http://www.whocc.no/atcddd/indexdatabase/index.php?query=N05CE) - N05CF [Benzodiazepine related drugs](http://www.whocc.no/atcddd/indexdatabase/index.php?query=N05CF) - N05CM [Other hypnotics and sedatives](http://www.whocc.no/atcddd/indexdatabase/index.php?query=N05CM) - N05CX [Hypnotics and sedatives in combination, excl. barbiturates](http://www.whocc.no/atcddd/indexdatabase/index.php?query=N05CX) |
| Other central nervous system (D211) | [**OTHER NERVOUS SYSTEM DRUGS**](http://www.whocc.no/atcddd/indexdatabase/index.php?query=N07) **[N07]**   - N07A [PARASYMPATHOMIMETICS](http://www.whocc.no/atcddd/indexdatabase/index.php?query=N07A)   - N07AA [Anticholinesterases](http://www.whocc.no/atcddd/indexdatabase/index.php?query=N07AA)   - N07AB [Choline esters](http://www.whocc.no/atcddd/indexdatabase/index.php?query=N07AB)   - N07AX [Other parasympathomimetics](http://www.whocc.no/atcddd/indexdatabase/index.php?query=N07AX) - N07B [DRUGS USED IN ADDICTIVE DISORDERS](http://www.whocc.no/atcddd/indexdatabase/index.php?query=N07B)   - N07BA [Drugs used in nicotine dependence](http://www.whocc.no/atcddd/indexdatabase/index.php?query=N07BA)   - N07BB [Drugs used in alcohol dependence](http://www.whocc.no/atcddd/indexdatabase/index.php?query=N07BB)   - N07BC [Drugs used in opioid dependence](http://www.whocc.no/atcddd/indexdatabase/index.php?query=N07BC) - N07C [ANTIVERTIGO PREPARATIONS](http://www.whocc.no/atcddd/indexdatabase/index.php?query=N07C) - N07X [OTHER NERVOUS SYSTEM DRUGS](http://www.whocc.no/atcddd/indexdatabase/index.php?query=N07X) |
| Urinary antispasmodics (D212) | [**Urinary antispasmodics**](http://www.whocc.no/atcddd/indexdatabase/index.php?query=G04BD) **[G04BD]** |
| Other specified drugs (D299(D299A) | Not described under D200-212.  D299A: Brief description (free text) |
| Surgery | |
| Cataract extraction (D300) | Surgical removal of a cataractous lens. |
| Pacemaker, artificial (D301) | A device designed to stimulate, by electric impulses, contraction of the heart muscles. It may be temporary (external) or permanent (internal or internal-external). |
| Podiatric surgery or intervention (D302) | Procedures performed by podiatric surgeons or podiatrists include a wide range of soft tissue and osseous procedures (e.g. correction of hammertoes and other digital deformities, ingrown toenail correction, hallux valgus / varus / limitus / rigidus correction, heel spur resection, plantar fasciectomy / fasciotomy, Morton's neuroma / nerve entrapment excision, cyst, ganglion and tumour excision, removal of foreign bodies, exostectomy, ligament repair, tendon lengthening, repair and transfer, insertion and removal of internal fixation). Also removal of callus, trimming of nails or provision of adapted footwear. |
| Others (D399/D399A) | Other surgeries not described under D300 & D301.  D399A: Brief description (free text) |
| Management of urinary incontinence (D400) | |
|  | e.g. (assisted toileting, bladder retraining, prompted voiding, pelvic floor exercises, antispasmodics) |
| Fluid or nutrition therapy (D500) | |
|  | - Fluid therapy whose basic objective is to restore the volume and composition of the body fluids to normal with respect to water-electrolyte balance. Fluids may be administered intravenously, orally, by intermittent gavage, or by hypodermoclysis. [MeSH D005440]. - Nutrition therapy: Improving health status of an individual by adjusting the quantities, qualities, and methods of nutrient intake [MeSH D044623]. |
| Psychological | |
| **Individual/Group** (D60B, D69B) | 1=individual,2=group,3=combination, 99=not described |
| Cognitive (behavioural) interventions (D600) | A direct form of psychotherapy based on the interpretation of situations (cognitive structure of experiences) that determine how an individual feels and behaves. It is based on the premise that cognition, the process of acquiring knowledge and forming beliefs, is a primary determinant of mood and behaviour. The therapy uses behavioural and verbal techniques to identify and correct negative thinking that is at the root of the aberrant behaviour [MeSH D015928]. |
| Others (D699/D699A) | Some other psychological approach is used and not described by D600.  D699A: Brief description (free text) |
| Environment/Assistive technology These categories are based on the International Standard ISO9999 “Technical aids for persons with disabilities – Classification and terminology”. | |
| ***Environment*** ***(Furnishings and adaptations to homes and other premises******ISO 9999:18) – direct action*** | |
| Dwelling unit, indoors incl. entrances (D700) | Furnishings and adaptations to homes and other premises [ISO 9999:18] that have been done and detailed in the article (e.g. report of compliance rate). Homes and other premises include private housing, institutions and sheltered houses. The intervention sites are indoors incl. entrances.  *Illustrative examples:*   - Light fixtures [ISO9999:1806] or “Lighting”: The illumination of an environment and the arrangement of lights to achieve an effect or optimal visibility [MeSH D008029]. - Sitting furniture [ISO9999:1809] (e.g. chaires which meet the specific seating requirements of a person) - Support devices [ISO9999: 1818] (e.g. Hand rails, support rails, grab-rails, hand-grips) - Beds [ISO9999: 1812] (e.g. fixed height and height-adjustable beds) - Gate, door, window and curtain openers, closers [ISO9999:1821] - Construction elements in the home and other premises: Features of the structure of the house which are designed to assist a disabled person to function independently [ISO9999:1824] - Vertical conveyors [ISO9999:1830] (e.g. stairlifts, ramps) - Safety equipment for the home and other premises [ISO9999:1833]) (e.g. non-slip materials for floors and stairs)   Aids for personal care and protection  *Illustrative examples:*   - Aids for toileting [ISO9999:0912] (e.g. commode chairs, raised toilet seats) - Aids for washing, bathing and showering [ISO9999:0933] (e.g. bath/shower chairs, non-slip bath mats, shower mats and tapes) |
| Dwelling unit, outdoors (D701) | Adaptations that have been done at outdoor areas belonging to private housing or institutions or sheltered houses and have been detailed in the article. |
| Public outdoor (D702) | Adaptations that have been done at public outdoor areas and have been detailed in the article.  *Illustrative examples:*   - pavement |
| Relocation (D703) | Changes of living arrangements like moving to assisted living or higher levels of formal care |
| ***Environment (Aids for personal mobility/ISO9999:12)*** | |
| Aids for personal mobility (D710) | *Providing the participants with aids for personal mobility if necessary.*  *Illustrative examples:*   - Walking aids manipulated by one arm [ISO9999:1203]   (e.g. walking-sticks, elbow crutches, forearm support crutches, axillary crutches, walking-sticks with three or more legs, walking-sticks with seat)   - Walking aids manipulated by both arms [ISO9999:1206]   (e.g. walking-frames, rollators, walking-chairs, walking tables)   - Accessories for walking-aids [ISO9999:1207] - Wheelchairs [ISO9999:1221] - Transfer aids: aids assisting change of position in relation to another activity [ISO9999:1230] (e.g. sliding boards, sliding mats, free standing rails for self lifting) - Lifting aids [ISO9999:1236] (e.g. mobile hoists with sling seats, standing mobile hoist, stationary hoists fixed to the wall, floor or ceiling), stationary free-standing hoists) |
| ***Environment (Aids for communication, information and signalling / ISO9999:21)*** | |
| Optical aids (D720) | *Illustrative Examples:*   - Eyeglasses: A pair of ophthalmic lenses in a frame or mounting which is supported by the nose and ears. The purpose is to aid or improve vision. [MeSH D005139] – or „Spectacle lenses“ [ISO9999:210303] - Contact lenses: Lenses designed to be worn on the front surface of the eyeball. [MeSH D003261] or “Contact lenses” [ISO9999:210309] |
| Hearing aids (D721) | - Hearing-aids (ISO9999: 2145)   *Illustrative Examples:*   - In-the ear hearing-aids: Devices worn within the ear to amplify sound; en-the-canal hearing-aids included [ISO9999: 214503] - Behind-the-ear Hearing-aids: Devices worn behind the ear to amplify sound; headband hearing-aids included [ISO9999: 214506] - Spectacle hearing-aids: Devices fixed onto a spectacle frame to amplify sound [ISO9999: 214509] - Body-worn hearing-aids [ISO9999: 214512] - Hearing-aids used in connection with implant: Electronic devices that stimulate the receptors in the inner ear [ISO9999: 214518] |
| Aids for signalling and indicating | *Illustrative Examples*   - Door-signals and door-signal indicators [ISO9999:214803] - Door-warners [ISO9999: 214806] - Indicators (Indicating devices applied to a product: e.g. Sensor mats) [ISO9999:214821] - Identification bracelets |
| Alarm systems (D723) | *Illustrative Examples*   - Personal emergency alarm systems: Alarm devices operated by the user [ISO9999: 215103] - Aids for emergency detection: Devices which automatically activate an alarm in case of emergency [ISO9999: 215109] - Monitoring systems: Devices to monitor the status of a specific situation [ISO9999:215115] |
| ***Environment (Body worn aids for personal care and protection)*** | |
| Body worn protective aids (D730) | Body worn equipment to prevent injury to parts of the body [ISO9999:0906]  *Illustrative examples:*   - Hip protectors |
| Clothes and shoes (D731) | Clothes and shoes [ISO9999:0903]  *Providing the participants with (anti-slip) clothes and shoes if necessary.*  *Illustrative examples:*   - Anti slip devices for shoes and boots [ISO9999:090345] - Stockings and socks [ISO9999: 090327] - Orthopaedic footwear (Footwear intended to treat and/or compensate for the structural or functional disorders of a person’s feet [ISO9999:0633] (Footwear provided in the context of podiatric interventions code D302 “Podiatric surgery and interventions”) |
| **Other environmental interventions (D799/D799A)** | Not described under D700-D731  D799A: Brief description (free text) |
| Environmental (social environment) | |
| Staff ratio (D800) | Changes in the number or contacts of formal caregivers |
| Staff training (D801) | Changes in the qualifications of   - formal caregivers - primary care physicians - primary care nurse - other involved health care professionals |
| Service model change (D802) | Change in the organisational system in which an intervention is delivered (e.g. clinic versus home), introduction of new health care models (e.g. chronic care clinic vs. usual primary care practice). |
| Telephone support (D803) | Regular telephone contact of health care professionals for informal and/or formal caregivers. |
| Caregiver training (D804) | Caregiver in this item includes only spouse and family caregivers. (Specific professional caregivers classify under 🡪 “Staff training” (D801))  Important aspects of fall prevention and safety at home should be targeted (e.g. exercise, environmental factors, nutrition, physical activity, home alarm, shoes and clothing, medication, transfers). The training should include next to general information also individual components.  Caregiver training can be delivered on an individual basis or in small groups.  (General recommendation and information given in lectures or written information classify under 🡪 “Written materials, videos, lectures” (D900)). |
| Home care services (D805) | Additional formal home care support |
| Others (D899/D899A) | Other social environmental interventions not described under D800-D805  D899A: Brief description (free text) |
| Knowledge | |
| Written materials, videos, lectures (D900) | This item is coded only for knowledge interventions that are not given to the control group as well.  *Illustrative examples:*   - pamphlets - information - booklets/sheets - videos - lectures |
| Others (D999/D999A) | Other interventions aiming to improve knowledge and not described by D900.  D999A: Brief description (free text) |
| Other interventions/procedures (D9999/D9999A) | Other interventions not described under D100 – D999.  D9999A: Brief description (free text) |
| Further specifications of the intervention | |
| ***Postintervention follow-up (D000)*** | |
| Postintervention follow-up (D000) | This describes whether there is any intention to prompt, review or encourage people with compliance or progression following the assessment and/or intervention period. For pharmacological studies code *post-intervention follow-up* if there are strategies like tablet counting or blood tests are made. (Don’t count any follow-up assessments targeting the outcome measures as post-intervention follow-up.) |
| Form (D00A) | 1=written, 2=by telephone, 3=personal, 4=combination, 99=not described |
| Number of contacts (D00B) | Total number of intended minimum contacts |
| Duration (D00C) | Postintervention period (months) |
| ***Strategies based on psychological models or theories to improve uptake and/or adherence (D010)*** | |
| Strategies based on psychological models or theories to improve uptake and/or adherence (D010) | Psychological approaches to increasing uptake would have the primary aim of increasing the probability that individuals would agree to take part in the trial, or try the intervention. There are numerous approaches that may be used but one might be to change the perception of the individual of the relevance of participation to him/herself another to make participation rewarding in some way. Theories that have been used in the past include Health Belief Model, Theory of Reasoned Action, Theory of Planned Behaviour, and Transtheoretical Model of Behaviour Change.  Such strategies focus e.g. on raising the awareness of the benefit taking part in the trial, encourage self management to improve adherence, a tailored approaches, social encouragement, promoting positive benefits which fits with a positive self identity. |
| ***Detailed description of the intervention available (D020)*** | |
| Detailed description of the intervention available (D020) | An intervention protocol or any precise description of the intervention is available (e.g. published, web link). |
| Availability (D020) | 1=directly from the author, 2=internet, 3=published, 4=others, 99=n.d. |

# Domain 4: Descriptors/Control Group

| Control Group | |
| --- | --- |
| Control group described (DC000) |  |
| Routine care/no specific interventions (DC100) | No special interventions for the control group |
| Supervised Exercises | |
| Exercises not targeted to increase mobility/lower limb function (DC210) |  |
| Others (DC299/DC299A) | Not described under DC210.  DC 299A: Brief description (free text) |
| Medication (Drug Target) | |
| Placebo (DC300) | Any dummy medication or treatment. Although placebos originally were medicinal preparations having no specific pharmacological activity against a targeted condition, the concept has been extended to include treatments or procedures, especially those administered to control groups in clinical trials in order to provide baseline measurements for the experimental protocol [MeSH D010919]. |
| Social environment/Knowledge | |
| Group sessions targeted to social interaction and support (DC400) | *Illustrative examples:*   - Social seminars - Discussion group |
| Telephone contacts (DC401) | *Illustrative examples:*   - Telephone contacts - Social home visits |
| Home visits (DC 402) | *Illustrative examples:*   - one-on-one friendly visits |
| Written materials, videos (DC403) | Information materials that have no focus on fall-prevention (e.g. information to general health topics) |
| Others (DC499/DC499A) | Not described under D400-D403)  DC499A: Brief description (free text) |
| Others (DC999/DC 999A) | Not classified under DC100-DC499.  DC999A: Brief description (free text) |

# References

- Technical aids for persons with disabilities - Classification and terminology: ISO9999 (2002), 3^rd^ edition, International Organization for Standardization, Geneva.
- International Standard Classification of Occupations: ISCO-88 (1990), International Labour Office (ILO), Geneva.
- Medical subject headings. MeSH Browser (2007) <http://www.nlm.nih.gov/mesh/MBrowser.html>, U.S. National Library of Medicine, Bethesda.
- Classification of Providers of Health Care (ICHA-HP). In **International Classification for Health Accounts 1.0** (2000). Organisation for Economic Co-operation and Development (OECD), Paris.
- ATC/DDD Index 2007. Internet source: <http://www.whocc.no/atcddd/>. WHO Collaborating Centre for Drug Statistics Methodology, Oslo.
- International Classification of Functioning, Disability and Health (ICF). Internet source: <http://www3.who.int/icf/icftemplate.cfm>. WHO, Geneva.
