# Supplementary material for: Reporting of complex interventions in clinical trials: development of a taxonomy to classify and describe fall-prevention interventions
Source: Trials. 2011 May 17;12:125. doi: 10.1186/1745-6215-12-125 (PMC3127768; doi:10.1186/1745-6215-12-125)
Supplement: Additional file 2 — Taxonomy to describe and conceptualise fall prevention interventions [file 1745-6215-12-125-S2.PDF]

Approach

Intervention group

PRIMARY AIMS

	(0=no,1=yes)	
To reduce falls		A100
To reduce fall related injuries		A101
To improve QOL		A102
To improve function/physical activity		A103
To reduce health/social care resource use		A104
Safety monitoring		A105
To improve psychological outcome		A106
Others		A199
Free text:...		A199A
Not described		A001

PRIMARY SELECTION CRITERIA

	(0=no,1=yes)	
**Population approach**		
**(none of the below except age)**		A200
**Selection criteria used**		
Demographics		
Age group (years)	≥	A300
Male only		A301
Female only		A302
Selected ethnic group		A303
Others		A399
Free text:		A399A
Previous falls (>=1)		A400
Chronic diseases, symptoms, impairments		
Osteoporosis/osteoporotic fractures		A500
Parkinson's disease/syndrome		A501
Cerebrovascular disorders		A502
Eye disorders, visual impairments		A503
Dementia, cognitive impairment		A504
Depression symptoms		A505
Syncope		A506
Gait and/or balance impairment		A507
Urinary Incontinence		A508
Screening tool		A509
Others		A599
Free text:		A599A
Medication-specific		A600
Specific groups excluded		
Dementia, cognitive impairment		A700
Other specified exclusion		A799
Free text:		A799A
No selection criteria specified		A002

Base

Intervention group

RECRUITMENT

Item	(0=no,1=yes)	Code
Hospitals		
Acute		B100
Emergency department		B101
Subacute (e.g. rehabilitation)		B102
**Nursing and residential care facilities**		B110
**Provider of ambulatory health care**		B120
**Community based**		B130
**Population based registers**		B140
**Others**		B199
Free text:		B199A
Not described		B001

MAIN SITE(S) OF DELIVERY

Item	(0=no,1=yes)	Code
Hospitals		
Acute		B200
Emergency department		B201
Subacute (e.g. rehabilitation)		B202
**Nursing and residential care facilities**		B210
**Provider of ambulatory health care**		B220
Community based		
Participant's home		B230
Organisations & other locations in the community		B231
Outdoor environment		B240
Others		B299
Free text:		B299A
Not described		B002

ASSESSMENTS DELIVERED BY

Item	(0=no,1=yes)	Code
Professionals		B300
Trained non-professionals		B301
Self-assessment		B302
Others		B399
Free text::		B399A
Not described		B003

INTERVENTIONS DELIVERED BY

Item	(0=no,1=yes)	Code
Professionals		
Medical doctors/medical assisstants		B400
Pharmacists		B401
Nursing and midwifery professionals		B402
Social work professionals		B403
Psychologists		B404
Physiotherapists & related professionals		B405
Occupational therapists & related professionals		B406
Modern health professionals not elsewhere classified		B407
Unspecified multidisciplinary/research teams		B408
Other professionals		B499
Free text:		B499A
Trained non-professionals		
Formal (e.g. students, paid personnel)		B500
Informal (e.g. volunteers, family members)		B501
Other non-professionals		B599
Free text:		B599A
**Self management interventions**		B600
**Institutions/authorities**		B700
**Others**		B999
Free text:..		B999A
Not described		B004

Components

Intervention group ☐

ASSESSMENTS AS PART OF THE INTERVENTIONS (including recommendations, referrals)

	(0=no,1=yes)	
Generic		
Geriatric Assessment (e.g. MDS-RAI)	☐	C100
Specific		
Validated fall risk assessment (e.g. PPA)	☐	C200
Gait and balance (only)	☐	C201
Cardiovascular assessment	☐	C202
Medication review	☐	C203
Vision	☐	C204
Foot assessment	☐	C205
Psychological assessmet	☐	C206
Environment (dwelling units) (e.g. Housing Enabler)	☐	C207
Environment (public outdoor)	☐	C208
Environment (aids for personal care and protection)	☐	C209
Others	☐	C299 Free text: C299A

COMBINATION OF INTERVENTIONS

	(0=no,1=yes)	
Single (single intervention)	☐	C300
Multiple (standardized combination)	☐	C301
Multifactorial (individual combination)	☐	C302

Descriptors

Intervention group [ ]

PROCEDURES & INTERVENTIONS

Supervised Exercises

Type of exercise	(0=no,1=yes)	**Duration** months 99=n.d.	**Frequency** per month 99=n. d.	**Intensity** 1=low, 2=moderate 3=hard, 99=n.d.	**Individual/Group** 1=individ., 2=group 3=combination 99=n.d.
Gait, balance, co-ordination, functional tasks	D100				
Strength/resistance (incl. power)	D101				
Flexibility	D102				
3D (tai chi, qi gong, dance, yoga)	D103	- D10A	D10B	D10C	D10D
General physical activity	D104				
Endurance	D105				
Other kind of exercises	D109	Free text: D109A			

Unsupervised Exercises

Type of exercise		**Duration** months 99=n.d.	**Frequency** per month 99=n. d.	**Intensity** 1=low, 2=moderate 3=hard, 99=n.d.	**Individual/Group** 1=individ., 2=group 3=combination 99=n.d.
Gait, balance, co-ordination, functional tasks	D1A0				
Strength/resistance (incl. power)	D1A1				
Flexibility	D1A2				
3D (tai chi, qi gong, dance, yoga)	D1A3	- D1AA	D1AB	D1AC	D1AD
General physical activity	D1A4				
Endurance	D1A5				
Other kind of exercises	D1A9	Free text: D1A9A			

Medication (drug target)

Direct action (withdrawal, dose reduction or increase, substitution, provision)

Antihypertensives	D200	Anti-dementia drugs	D207
Other cardiovascular agents	D201	Antidepressants	D208
Vitamin D	D202	Antipsychotic/Neuroleptic drugs	D209
Calcium	D203	Anxyolitics, hypnotics&sedatives	D210
Other bone health medication	D204	Other central nervous system	D211
Drugs used in diabetes	D205	Urinary antispasmodics	D212
Anti-Parkinson drugs	D206		
Other specified drugs	D299	Free text:	D299A

Surgery

Cataract	D300	
Pacemaker	D301	
Podiatric surgery	D302	
Others	D399	Free text: D399A

Management of urinary incontinence

D400

Fluid or nutrition therapy

D500

Psychological

			**Indivudual/Group** 1=individual,2=group, 3=combination, 99=n.d.
Cognitive (behavioral) interventions	D600		D60B
Others......................................	D699	Free text: D499A	D69B

Environment/Assistive technology

Furnishings and adaptations to homes and other premises/direct action

(0=no,1=yes)

Dwelling unit, indoors incl. entrances [ ] D700

Dwelling unit, outdoors [ ] D701

Public outdoor [ ] D702

Relocation [ ] D703

Aids for personal mobility [ ] D710

Aids for communication, information and signalling

Optical aids [ ] D720

Hearing aids [ ] D721

Aids for signalling & indicating [ ] D722

Alarm systems [ ] D723

Body worn aids for personal care and protection

Body worn protective aids [ ] D730

Clothes and shoes [ ] D731

Other environmental interventions [ ] D799 Free text: D799A

Social environment

Staff ratio [ ] D800

Staff training [ ] D801

Service model change [ ] D802

Telephone support [ ] D803

Caregiver training [ ] D804

Home care services [ ] D805

Others .... [ ] D899 Free text:...................... .... D899A

Knowledge

Written material, videos, lectures etc. [ ] D900

Others .... [ ] D999 Free text: D999B

Other Interventions

[ ] D9999 Free text:..D9999A

FURTHER SPECIFICATIONS OF THE INTERVENTIONS

		1=written, 2=by telephone, 3=personal,99=n.d.	Number of contacts	Postintervention period (months)
**Postintervention follow-up**	[ ] D000	[ ] D00A	[ ] D00B	[ ] D00C

Strategies based on psycho-logical models or theories to improve uptake and/or adherence [ ] D010

Model/Theory

Free text: D010A

Detailed description of the intervention available [ ] D020

1=directly from the author, 2=internet, 3=published, 4=others, 99=n.d. [ ] D02A

Descriptors

UNSPECIFIC CONTROL GROUP / SHAM INTERVENTION

(0=no,1=yes)

Control group described ☐ DC000

Routine care,no specific interventions ☐ DC100

Supervised Exercises

Exercises not targeted to increase mobility or lower limb function ☐ DC210

Others .. ☐ DC299 Free text: DC299A

Medication(Drug Target)

Placebo ☐ DC300

Social environment/Knowledge

Group sessions ☐ DC400
Telephone contacts ☐ DC401
Home visits ☐ DC402
Written materials, videos ☐ DC403

Others ☐ DC499 Free text: DC499A

Others ☐ DC999 Free text: DC999A
